# Large-scale proteomic analysis of the grapevine leaf apoplastic fluid reveals mainly stress-related proteins and cell wall modifying enzymes

**DOI:** 10.1186/1471-2229-13-24

**Published:** 2013-02-08

**Authors:** Bertrand Delaunois, Thomas Colby, Nicolas Belloy, Alexandra Conreux, Anne Harzen, Fabienne Baillieul, Christophe Clément, Jürgen Schmidt, Philippe Jeandet, Sylvain Cordelier

**Affiliations:** 1 Université de Reims Champagne-Ardenne, UFR Sciences Exactes et Naturelles, Unité de Recherche Vigne et Vins de Champagne – EA 4707, Laboratoire d’Œnologie et de Chimie Appliquée, B.P. 1039, Reims, cedex 02, 51687, France; 2 Université de Reims Champagne-Ardenne, UFR Sciences Exactes et Naturelles, Unité de Recherche Vigne et Vins de Champagne – EA 4707, Laboratoire de Stress, Défenses et Reproduction des Plantes, B.P. 1039, Reims, cedex 02, 51687, France; 3 Max-Planck-Institute for Plant Breeding Research, Mass Spectrometry Group, Carl-von-Linné-Weg 10, Köln, D-50829, Germany; 4 Université de Reims Champagne-Ardenne, UFR Sciences Exactes et Naturelles, Laboratoire de Signalisation et Récepteurs Matriciels (SiRMa), UMR CNRS 6237, Plate-forme de Modélisation Moléculaire, B.P. 1039, Reims, cedex 02, 51687, France

**Keywords:** Apoplastic fluid extraction, 2D electrophoresis, Mass spectrometry, Proteomic map, *Vitis vinifera*

## Abstract

**Background:**

The extracellular space or apoplast forms a path through the whole plant and acts as an interface with the environment. The apoplast is composed of plant cell wall and space within which apoplastic fluid provides a means of delivering molecules and facilitates intercellular communications. However, the apoplastic fluid extraction from *in planta* systems remains challenging and this is particularly true for grapevine (*Vitis vinifera* L.), a worldwide-cultivated fruit plant. Large-scale proteomic analysis reveals the protein content of the grapevine leaf apoplastic fluid and the free interactive proteome map considerably facilitates the study of the grapevine proteome.

**Results:**

To obtain a snapshot of the grapevine apoplastic fluid proteome, a vacuum-infiltration-centrifugation method was optimized to collect the apoplastic fluid from non-challenged grapevine leaves. Soluble apoplastic protein patterns were then compared to whole leaf soluble protein profiles by 2D-PAGE analyses. Subsequent MALDI-TOF/TOF mass spectrometry of tryptically digested protein spots was used to identify proteins. This large-scale proteomic analysis established a well-defined proteomic map of whole leaf and leaf apoplastic soluble proteins, with 223 and 177 analyzed spots, respectively. All data arising from proteomic, MS and MS/MS analyses were deposited in the public database world-2DPAGE. Prediction tools revealed a high proportion of (i) classical secreted proteins but also of non-classical secreted proteins namely Leaderless Secreted Proteins (LSPs) in the apoplastic protein content and (ii) proteins potentially involved in stress reactions and/or in cell wall metabolism.

**Conclusions:**

This approach provides free online interactive reference maps annotating a large number of soluble proteins of the whole leaf and the apoplastic fluid of grapevine leaf. To our knowledge, this is the first detailed proteome study of grapevine apoplastic fluid providing a comprehensive overview of the most abundant proteins present in the apoplast of grapevine leaf that could be further characterized in order to elucidate their physiological function.

## Background

Grapevine (*Vitis vinifera* L.) is one of the most cultivated fruit plants worldwide with an important economic impact due to the high value of its derivative products such as grapes and juice, wine and liquors. Since the availability of its genome sequence, grapevine was established as a non-climacteric model plant [1,2].

The apoplast is defined as the total extracellular space external to the plasma membrane [3]. The fluid moving in the extracellular space is usually named apoplastic fluid (AF). It contains a large variety of molecules that are known to be involved in various processes, including (i) growth regulation, (ii) cell wall maintenance, (iii) protection against desiccation and environmental stresses, (iv) transportation route for a broad range of molecules, (v) homeostasis, (vi) cell to cell adhesions and (vii) gas exchanges (for review see [4]). It plays a crucial role in plant defence mechanisms because it provides a continuous network in plants, representing the interface between the plant and its environment [5,6]. Despite their biological significance, investigations on apoplastic proteins are hampered due to their low abundance compared to intracellular protein concentrations. Moreover, the AF extraction from *in planta* systems is far from easy and remains challenging. The limited number of studies that have been performed on *in planta* secretomes is explained by the requisite optimization of existing protocols for each plant species. This is particularly true for a recalcitrant plant like grapevine regarding its polyphenols and polysaccharides contents. The most commonly used method for AF extraction is the vacuum-infiltration-centrifugation (VIC) method involving two critical steps: vacuum-infiltration with appropriate extraction buffer and centrifugation [3,7]. The VIC method has been used for AF protein recovery and shown to be suitable for proteomic analyses. 2D-PAGE is known to be a robust global approach to obtain a snapshot of the secretome [3,8]. Since the accomplishment of the *V. vinifera* genome sequencing, genome scanning using dedicated software allowed numerous protein predictions [1]. However, their presence has still to be confirmed and the use of the public databases improved considerably the potential of grapevine proteome analysis [9].

The present work aimed at evaluating the potential of subcellular proteomics for identifying proteins in the apoplastic compartment of grapevine leaf. Subcellular proteomics has the advantage not only of relating proteins to a functional compartment of eukaryotic cells, but also of reducing the complexity of the tissue protein extracts, which often prevents a satisfactory proteomic analysis. Indeed, as the resolution of analytical separation methods is too limited to dissect the total proteome of a cell or tissue, less abundant proteins are often masked by those expressed at higher levels [10]. In this study, we present an optimized VIC method to extract soluble proteins from AF of grapevine leaves and a well-defined interactive map of these proteins. Improvement of this method was carried out by comparing apoplastic to whole leaf soluble protein extracts. Large-scale analysis of the grape AF revealed that stress-related proteins and cell wall modifying enzymes are predominately present in this cellular compartment.

## Results and discussion

### Apoplastic fluid recovery and protein extraction optimization

In order to obtain a large overview of the proteins naturally present in the AF of non-stressed grapevine, a large-scale proteomic study by 2D-PAGE analyses has been realized. In grapevine, like most plants, the large amount of intracellular proteins, among which the RuBisCo is most highly represented, doesn’t allow to specifically identify AF proteins from total protein extracts. A VIC method (for review see Lohaus *et al.*, 2001 [7]) has been optimized from results obtained in barley [11] and tomato [12] to recover the AF of grapevine leaf and to obtain a protein sample enriched in apoplastic proteins. The AF of grapevine leaf was difficult to recover by centrifugation. To enhance AF outflow, a major modification of the usual VIC method was to increase centrifugation speed up to 7,500 g. Such centrifugation may cause damage to cell walls and membranes leading to contamination of the AF by inner cell components [7]. However, neither malate dehydrogenase nor NH4^+^, both cytoplasmic markers, have been reported in the *Brassica napus* leaf AF, which was recovered after a 12,000 g centrifugation step [13]. Protein contaminants from other subcellular compartments are often found in AF and, depending on the extraction method, slightly different results on recovered proteins are expected [14-19].

**Figure 1:**
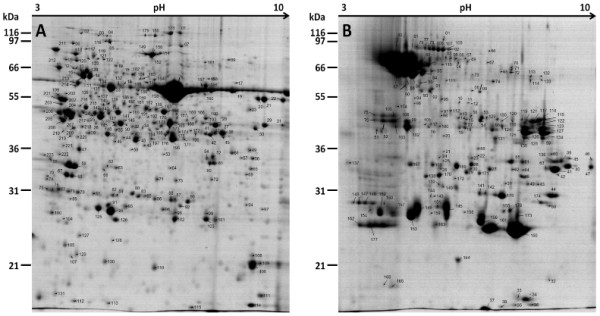
**2-DE Coomassie blue stained gels from whole leaf proteome (A) and apoplastic fluid (B) of *V. vinifera *leaves*.*** Protein extracts were separated on 18 cm, pH 3–10 non-linear gradient IPG gels (IEF) followed by 12% SDS PAGE. Arrows show the position of the analyzed spots. The protein spot number refers to Additional file 1 for whole leaf proteins and to Additional file 2 for apoplastic proteins. White arrows show examples of common proteins found in gel B (RuBisCo subunit (09), ATPase (90) and sedoheptulose-1,7 bisphosphatase (52)) and their respective spot in gel A.

During protein extraction with TCA/acetone buffer, the precipitation of sugars present in the AF sample leads to a viscous extract not suitable for IEF. To clean up proteins from leaf AF before further processing, a procedure with TCA/acetone combined to a phenol extraction step was performed. Denatured proteins and other hydrophobic proteins are soluble in phenol or agglomerate at the phenol-water interface, unlike the small molecules and nucleic acids, which are soluble in the aqueous phase. The combined use of TCA/acetone precipitation and phenol-based extraction improved sample quality, resulting in a better spot resolution, as reflected in reduced background and streaking on 2-D electrophoresis gels and leading to a high number of detected spots (Figure 1).

### Protein identifications

Protein separation by 2D-PAGE and MS identification were performed in order to compare the protein profiles obtained from whole leaf (Figure 1A) and AF (Figure 1B), as reported by previous work [8,20]. Two clearly different protein patterns were obtained by 2D-PAGE, demonstrating an apparent subfractionation of the AF. Comparative analysis of the acquired gel images from 4 biological replicates was carried out using the Delta2D software. The analyses revealed a total of 448 spots in the whole leaf extract and 306 spots in the AF extract over a non-linear pH range of 3–10 and a size range of 15–120 kDa. Respectively 223 and 177 spots were analyzed by MS and MS/MS in order to construct whole leaf and apoplast 2D maps of *V. vinifera*. MS analyses yielded respectively 227 and 89 different positive identifications of proteins and their respective isoforms.

Data arising from gel analyses are deposited in the public database World-2DPAGE (accession number 0037-Mapping the proteome and secretome of *Vitis vinifera* leaves) [21]. The corresponding proteins are also listed in the Additional file 1 for whole leaf proteins and in Additional file 2 for apoplastic proteins. Although the *V. vinifera* genome was recently sequenced [1], little is known about the protein profiles of grapevine. Numerous identifications were based on homology to proteins characterized in other species by Blast-p search of the NCBI non-redundant database [22]. These identifications lead to the *in planta* detection of 66 *V. vinifera* predicted proteins, confirming their presence in grapevine leaf AF (Table 1).

**Table 1:** Annotation of the proteins in the grapevine apoplastic fluid leaf

**Classes**^**a**^	**Subclasses**^**b**^	**Spots n°**^**c**^	**Gi acc. n°**^**d**^	**Loc.**^**e**^	**Closer homologous protein by Blastp [species]**^**f**^	**% hom.**^**g**^
PR proteins	chitinases	19;55;127;134;135	157329111	C	chitinase, putative [Ricinus communis]	75
		31	15213852	S	chitinase-B [Sorghum halepense]	100
		44	225462669	S	class III chitinase [Vitis vinifera]	99
		55;59;121;122;125;127;128;134;135	225469348	S	chitinase, putative [Ricinus communis]	77
		65	10880381	S	chitinase [Vitis vinifera]	100
		140;141;175	116333	S	chitinase B [Zea mays]	100
		142	147820457	S	chitinase 1 precursor, putative [Ricinus communis]	76
		144	266324	NC	chitinase-B [Sorghum halepense]	62
		147;148;149	225454408	S	acidic chitinase III [Nicotiana tabacum]	82
		155	225434076	C	class IV chitinase [Vitis pseudoreticulata]	87
		176	157353734	C	class IV chitinase [Vitis pseudoreticulata]	87
	glucanases	136	163914215	S, TM	beta 1–3 glucanase [Vitis hybrid cultivar]	100
		39;40;45;46;47;63;136	225441375	S, TM	beta 1–3 glucanase [Vitis vinifera]	99
		24;41;42;61;64;136	225441379	S	beta-1,3-glucanase [Vitis riparia]	99
		27;167;168;169	225441373	S, TM	beta-1,3-glucanase [Vitis riparia]	86
		22	170243	S, TM	beta-1,3-glucanase [Vitis vinifera]	84
	other PR proteins	35	225429115	S	pathogenesis-related protein 1 [Vitis hybrid cultivar]	100
		36	225429250	S	pathogenesis-related protein 1 [Vitis hybrid cultivar]	88
		37	225429119	S	putative pathogenesis-related protein 1 [Vitis hybrid cultivar]	98
		33;34;36;49;160;161;163;173	225426801	S, TM	osmotin-like protein [Vitis vinifera]	90
		177	7406714	M	putative thaumatin-like protein [Vitis vinifera]	100
		162;177	225426793	S, TM	thaumatin-like protein [Vitis vinifera]	99
		**145;146;150;151;156**	**225444754**	**S**	**germin-like protein 6 [Vitis vinifera]**	**100**
		152;153;154;157;159	225429295	S	NtPRp27 [Nicotiana tabacum]	83
		166	147853970	UC	pathogenesis-related protein 10.7 [Vitis vinifera]	73
		164	225453020	S	Wound-induced protein WIN2 precursor, putative [Ricinus communis]	82
proteases	serine proteinases	83	225449346	NC	cucumisin precursor, putative [Ricinus communis]	76
		**3;80;81;82;83;89;90;94;95;119**	**157344189**	**NC**	**cucumisin precursor, putative [Ricinus communis]**	**78**
		68;70;71;72	157335112	NC	subtilisin-type protease precursor [Glycine max]	80
		69	1771160	S	xylem serine proteinase 1 precursor, putative [Ricinus communis]	85
		110	157335622	NC	xylem serine proteinase 1 precursor, putative [Ricinus communis]	85
		4;5	157345245	S, TM	xylem serine proteinase 1 precursor, putative [Ricinus communis]	89
		111	225457767	S	serine carboxypeptidase, putative [Ricinus communis]	88
		172	157348245	S, TM	serine carboxypeptidase, putative [Ricinus communis]	79
	aspartic proteinases	48;115;117;118;119;123;126	157335788	M	aspartic proteinase nepenthesin-1 precursor, putative [Ricinus communis]	85
		14;18;129	157339844	S	aspartic proteinase nepenthesin-2 precursor, putative [Ricinus communis]	75
		15;16;17;57;99;102;103;120;124	225430555	S	aspartic proteinase nepenthesin-2 precursor, putative [Ricinus communis]	76
cell wall metabolism	hydrolases	28;29;171	147771556	S, TM	xyloglucan endotransglucosylase/hydrolase [Gossypium hirsutum]	90
		67	157354845	S, TM	glycosyl hydrolase family 38 protein [Arabidopsis thaliana]	77
	glucosidases	1	147821903	S, TM	alpha-glucosidase, putative [Ricinus communis]	87
		66	147787240	S	alpha-glucosidase, putative [Ricinus communis]	79
		2;77;78;79;107;108;109	225423961	S, TM	alpha-glucosidase, putative [Ricinus communis]	90
		84;85	157350003	S	beta-glucosidase, putative [Ricinus communis]	90
		84;85;86;87;88;96	157355824	S, TM	beta-glucosidase, putative [Ricinus communis]	90
	galactosidases	101	147810287	UC	alpha-galactosidase/alpha-n-acetylgalactosaminidase, putative [Ricinus communis]	92
		58	157329180	NC	beta-galactosidase, putative [Ricinus communis]	87
		13;50;53;56;98;174	157337481	S, TM	beta-galactosidase, putative [Ricinus communis]	89
		97;112	157332401	S	beta-galactosidase, putative [Ricinus communis]	88
	fucosidase	15;53;54	225424647	S, TM	alpha-L-fucosidase 2 precursor, putative [Ricinus communis]	82
	arabinofuranosidase	3;6;7	225440254	S, TM	alpha-N-arabinofuranosidase 1 precursor, putative [Ricinus communis]	83
	polygalacturonase	81	225426168	S, TM	polygalacturonase, putative [Ricinus communis]	88
	pectinesterases	43;49	157353359	NC	pectinesterase-2 precursor, putative [Ricinus communis]	71
		56;130	157350105	S, TM	pectinesterase-3 precursor, putative [Ricinus communis]	78
		73	157353908	S	pectin methylesterase 2 [Pyrus communis]	86
peroxidases		23;26;29;62	225459180	S	cationic peroxidase [Arachis hypogaea]	85
		60	157355449	S, TM	class III peroxidase [Gossypium hirsutum]	86
		21;25	157355447	S, TM	class III peroxidase [Gossypium hirsutum]	89
		20;75;76;105;106	225435616	S	peroxidase [Armoracia rusticana]	80
		131;158	223635592	NC	peroxidase 1 [Scutellaria baicalensis]	80
		131	157342951	S	peroxidase 25 precursor, putative [Ricinus communis]	83
other stress related proteins		165	134684	C	putative Cu/Zn superoxide dismutase precursor [Vitis vinifera]	83
		127	26224736	NC	serpin-like protein [Citrus x paradisi]	85
		113;114	147846526	S	reticuline oxidase precursor, putative [Ricinus communis]	80
		132;133	147825300	S, TM	reticuline oxidase precursor, putative [Ricinus communis]	82
		**21**	**76559894**	**UC**	**isoflavone reductase-like protein 5 [Vitis vinifera]**	**100**
		10;11;12	157360089	S	heparanase, putative [Ricinus communis]	84
		43	115488670	C	universal stress protein family protein, expressed [Oryza sativa Japonica Group]	100
micellaneous		138;139;170	225443264	S	acid phosphatase [Glycine max]	79
		104	147814943	S, TM	alpha-amylase [Vigna angularis]	81
		**90**	**91983977**	**UC**	**ATP synthase CF1 alpha subunit [Vitis vinifera]**	**100**
		**81**	**224365667**	**UC**	**ATPase alpha subunit [Vitis vinifera]**	**100**
		137	157343592	UC	chloroplast heat shock protein 70–2 [Ipomoea nil]	93
		**52**	**225466690**	**C**	**chloroplast sedoheptulose-1,7-bisphosphatase [Solanum lycopersicum]**	**95**
		32	225457957	UC	cyclophilin [Catharanthus roseus]	94
		124	225454430	S, TM	lanatoside 15′-O-acetylesterase [Digitalis lanata]	84
		83	157343878	S	lysosomal alpha-mannosidase, putative [Ricinus communis]	86
		20;92;93	157343877	UC	lysosomal alpha-mannosidase, putative [Ricinus communis]	83
		51;52;74	157341194	S	lysosomal alpha-mannosidase, putative [Ricinus communis]	87
		**143**	**225440390**	**NC**	**NAD-dependent epimerase/dehydratase [Zea mays]**	**92**
		**32;38**	**225453348**	**UC**	**nucleoside diphosphate kinase B [Jatropha curcas]**	**95**
		119	147769722	C	polyprotein [Oryza australiensis]	58
		100	11385598	M	putative chloroplast RNA helicase VDL’ isoform 2 [Nicotiana tabacum]	100
		8	149774708	NC	ribulose-1,5-bisphosphate carboxylase/oxygenase [Ophioglossum petiolatum]	100
		**9**	**147769051**	**UC**	**ribulose-1,5-bisphosphate carboxylase/oxygenase large subunit [Vitis vinifera]**	**94**
		**116**	**225436591**	**UC**	**serine-pyruvate aminotransferase, putative [Ricinus communis]**	**95**
		19	116830469	C	hypothetical protein [Arabidopsis thaliana]	100
		30	125563554	UC	hypothetical protein [Vitis vinifera]	49
		69	157343556	C	hypothetical protein [Vitis vinifera]	100
		91	147818959	NC	Ca/calmodulin-dependent protein kinase	40

^a^ the protein distribution according to their biological process.

^b^ the protein group.

^c^ the number that identifies protein spots on 2-D apoplastic map.

^d^ the accession number from GenBank assigned to the peptide after MS/MS analysis.

^e^ the predicted localisation of the peptide according to the presence of a N-terminal signal peptide (S), non-classical signal peptide (NC), transmembrane helices (TM), chloroplast (C), mitochondria (M) and unclassified (UC) location.

^f^ the peptide identification based on homology to proteins characterized in other species by Blast-p search on NCBI database.

^g^ the relative homology percentage of protein obtained by Blast-p.

Shared proteins identified in both AF sample and total leaf sample are highlighted in bold.

Protein p*I* and *M*_r_ values were experimentally determined and compared to their theoretical values obtained from primary structure analysis (Additional file 1 and 2). Some theoretical *M*_r_/p*I* values did not match well compared to their experimental values on 2D maps. Such differences could be explained by possible post-translational modifications (PTMs) that cause the covalent addition of chemical groups to the polypeptide backbone [23]. The p*I* and *M*_r_ shifts might also be due to the removal of N-terminal signal peptides of secreted proteins, which leads to smaller *M*_r_ and p*I* modifications compared to the predicted sequences. Numerous spots matched the same protein accession. Peptide redundancies in different spots were probably due to PTMs. Moreover, in some spots, peptide mixtures could occur due to co-migration of proteins sharing the same p*I* and *M*_r_.

### Quality of the AF sample enrichment

**Figure 2:**
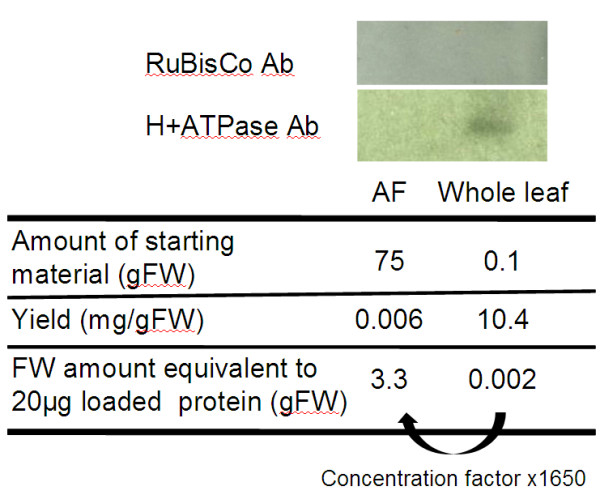
**H**^**+**^**-ATPase and RuBisCo large subunit are not detected in AF sample.** The upper panel shows the western blot analysis performed on 20 μg of protein from AF and whole leaf samples using anti-RbcL (RuBisCo Ab) and anti-H^+^-ATPase (H+ATPase Ab) antibodies. The bands corresponding to the H^+^-ATPase and RuBisCo large subunit are only detected in the whole leaf sample. The lower table indicates the yield of protein extraction expressed in mg of protein per g of FW and the equivalent of loaded FW per 20 μg of protein.

The cytoplasmic localization of malate dehydrogenase leads to its activity being used as a common marker of AF quality: the absence of activity in the AF argues for no or little cytoplasmic contamination [24]. Since the extraction buffer contains CHAPS denaturing agent, enzymatic activities could not be performed on the AF sample. However, no malate dehydrogenase was found among the analyzed spots in the AF in contrast to the whole leaf proteome where malate dehydrogenase is frequently detected (see spot n° 24, 44, 51, 173, 174, 177 in Figure 1A). The lack of the malate dehydrogenase in the AF sample suggests an enrichment in apoplastic proteins putatively without any contamination by cytoplasmic proteins. Nevertheless, Western-blot analysis has been performed on both AF and whole leaf samples to check the contamination level by other compartment proteins and to confirm the sample enrichment in AF proteins (Figure 2). The soluble proteins extraction yield reached approximately 6 μg and 10,400 μg per gram of fresh weight for AF sample and whole leaf sample, respectively. By loading the same amount of proteins, the loaded amount in equivalent fresh weight is around 1,650 times higher for AF sample than whole leaf sample (Figure 2). Specific antibodies of RuBisCo, the most abundant protein found in plant leaves, mainly localised in cytoplasmic and chloroplastic compartments [25] and H^+^-ATPase, a plasma membrane protein [26], have been used as markers of the AF quality to detect the contamination level. The RuBisCo large subunit and the H^+^-ATPase are present in the whole leaf protein sample as expected but are not detected in the AF protein sample (Figure 2). Even by loading 1,650 times more sample in equivalent fresh weight, neither the RuBisCo large subunit (cytoplasmic marker) nor the H^+^-ATPase (membrane marker) could be detected by Western-blot analysis in the AF sample. However, the detection by 2D-gel analysis of the RuBisCo subunit (spot 09, Figure 1B) and the ATPase (spot 90, Figure 1B) in AF sample indicates a very low level of symplastic contamination (Table 1 and Figure 1B). This contamination can be detected by the proteomic approach since 250 μg of protein is loaded onto the 2D-gels compared to the 20 μg loaded onto the Western-blot analysis. Some other cytoplasmic proteins have been extracted from the AF, as revealed by the presence of the chloroplastic sedoheptulose-1,7 bisphophatase (spot n° 52, Figure 1B) and suggesting a partial cell membrane breakdown. This recurrent and unavoidable cytoplasmic contamination has already been highlighted by several previous studies [8,14-19]. However, in such conditions, only 10 proteins highlighted in Table 1 could be identified in the whole leaf extract. This redundancy could be due to (i) cell component contamination as mentioned previously, such as the RuBisCo large subunit or ATPase, (ii) the high content of some of these apoplastic proteins, which can be significantly recovered by whole leaf extraction, such as the germin-like protein or cucumisin precursor. These data confirm that the VIC technique seems to be a suitable method allowing the recovery of a highly enriched AF protein sample from grapevine leaf.

### Global analysis of whole leaf and apoplast proteomes

**Figure 3:**
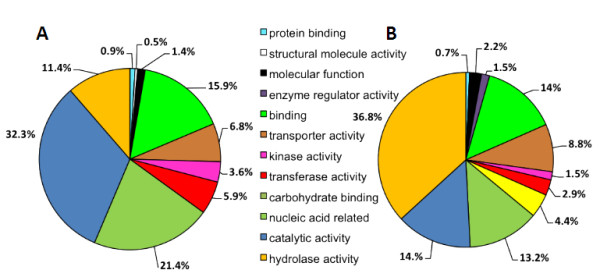
**Whole leaf (A) and apoplastic (B) protein classification on the basis to the molecular function they belong according to their GO annotation.** “Nucleic acid-related” corresponds to the merged classes: nucleic acid binding, RNA binding, DNA binding, chromatin binding, nucleotide binding, nuclease activity and translation factor activity.

Identified proteins in both apoplastic and whole leaf protein extracts were classified according to their molecular function (Figure 3). The GO database (http://www.geneontology.org) provides a useful tool to annotate and analyze the functions of a large number of proteins. Categories were based on GO classification using AgBase, a unified resource for functional analysis in agriculture [27]. Proteins were grouped according to plant GO-slim categories obtained for molecular functions. Some of the GO classes were merged in order to simplify the classification.

The molecular function classification covered a higher proportion of peptides with hydrolase activity (36.8%) and carbohydrate binding pattern (4.4%) in the AF extract compared to the whole leaf extract (11.4 and 0% respectively). The latter showed predominantly peptides annotated to catalytic activity (32.3%) and nucleic acid related (21.4%). The high rate of peptides with hydrolase activity and/or involved in carbohydrate binding could be linked to the numerous glycosyl hydrolase and pathogenesis-related (PR) proteins found in the AF (Table 1). The higher proportion of peptides in the whole leaf protein extracts that could interact with nucleic acids supports the conclusion that they are localized in the cytoplasm, therefore suggesting the quality of the AF enrichment process.

### Protein secretion

**Figure 4:**
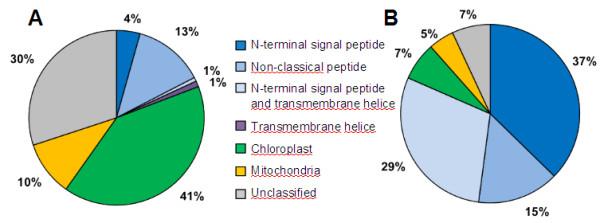
**Whole leaf (A) and apoplastic (B) protein distribution according to the presence of a peptide signal and consensus region.** N-terminal signal peptide (S), non-classical signal peptide (NC), transmembrane helices (TM), chloroplast (**C**) and mitochondria (M) location. Analyses were performed with TargetP (S, C, M), SecretomeP (NC) and TMHMM server v.2.0 (TM). UC represents the unclassified proteins.

From the AF proteins, 66% were predicted with a signal peptide sequence using the TargetP software [28], compared to 5% for the whole leaf proteins (Figure 4, Additional files 1 and 2). A similar percentage of proteins with a predicted signal peptide sequence was found in soybean (65%), but lower amounts were found in Arabidopsis (47%) and rice (37%) [19,29,30]. Predicted cleavage sites within the N-terminal region of most proteins identified in the AF are additional support for the quality of the AF extraction. In the classical secretory pathway, proteins are synthesized in the endoplasmic reticulum before passing through the dictyosomes to be inserted by vesicles in the plasma membrane or secreted into the extracellular space. Among the secreted proteins, some can be linked to the cell membrane. An analysis with the TMHMM algorithm [31,32] confirmed that 29% of the proteins in the AF extract harbour at least one α-helical transmembrane domain compared to the whole leaf extract in which only 2% of identified proteins are transmembrane proteins (Figure 4). This difference could suggest either a putative rupture of the cytoplasmic membrane leading to the release of these proteins in the apoplast and to a cytoplasmic contamination of the AF sample or the secretion of these proteins through the cytoplasmic membrane. Indeed, proteins identified in the AF and lacking a signal peptide, named leaderless secreted proteins (LSPs), could be secreted by a non-classical secretory mechanism as described in yeast and bacteria [3,33-35]. Inventories of plant secretome reveal that LSPs may account for up to 50% of the whole leaf proteins identified in the extracellular fluid [3]. LSPs potentially possess dual functions depending on the localization inside or outside plant cells. Part of the predicted cytoplasmic proteins found in the AF could be in fact actively translocated into the extracellular space. They could have another function such as the heat shock protein 70 (spot n° 137, Figure 1B), which is thought to be secreted in maize after pathogen elicitation [36]. The presence of LSPs can be predicted by a SecretomeP analysis [34]. Data analyses revealed that the protein sample recovered by the VIC method contains 15% of LSPs (Figure 4). Such a low level could be explained by the fact that analyses were performed on unstressed leaves. The secretome of plants submitted to stresses usually contains more LSPs than unstressed plants. Indeed, the analysis of *Arabidopsis* salicylic acid-treated cells reveals that more than 55% of the secreted proteins were LSPs [37].

Altogether the chloroplastic, mitochondrial and transmembrane proteins represent 52% of the whole leaf extract and only 12% of the AF extract (Figure 4). The amount of contamination in the AF by cellular components can be observed especially with the minimal content of chloroplastic and mitochondrial proteins. These intracellular protein contaminations have already been described in previous reports and are consistent with the proposed occurrence of non-classical secretory pathways [8]. Notably, the prediction of cellular localization and signal peptide content enabled us to conclude that the VIC method developed here was suitable for an enrichment of AF proteins.

### Apoplastic fluid proteins analysis reveals mainly stress-related proteins, proteases and cell wall modifying enzymes

Apoplastic proteins were classified in functional groups according to their identification (Table 1). Most of the proteins found in the AF can be related to plant defence mechanisms or cell wall metabolism. Among the analysed proteins listed in Table 1, 26 different PR proteins and 17 cell wall metabolism proteins were identified representing respectively 28% and 18% of classified proteins in grapevine leaf AF. This protein distribution is quite similar to those observed in oilseed rape, rice, alfalfa or poplar [16,20,24,29]. However, in the AF of maize, the proteins related to cell wall metabolism seem to be over represented with 58% of identified proteins compared to the 22% of defence-related proteins [8]. In contrast, the defence-related proteins in AF of tobacco reach 45% of total identified proteins [38].

**Figure 5:**
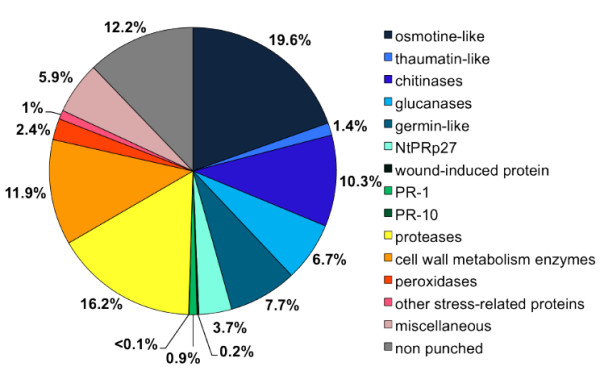
**Relative abundance on 2-D gels of proteins functional groups identified in the apoplastic fluid.** Values represent the percentage of the spot volume sums for all proteins of the functional group relative to the overall AF proteins.

The number of protein identification does not represent the relative quantity of the proteins. We estimated the quantity of each identified protein from the respective spot area on the 2D gel. The spot volume averages from the 4 biological repetitions were reported for each functional group. If co-migrations and multiple identifications were found in one spot, then peptides arbitrarily shared the spot quantity. Figure 5 displays the distribution by relative quantity of each functional group of AF proteins. Thus, PR proteins represent 50.7% overall of the apoplastic protein contents on 2-D gels; proteases, 16%; cell wall modifying enzymes, 11.9%; peroxidases 2.4% and 1% could also be linked to a stress response. The remaining 12.2% of the apoplast proteins, in terms of relative protein content on 2-D gel, were not analyzed. These figures emphasize even more the part of defence related proteins in the grapevine leaf AF, as it can be observed in tobacco [38] whereas it is rather the cell wall metabolism related proteins that are the most represented in poplar or maize [8,20].

#### Pathogenesis-related proteins

PR proteins have a crucial role in plant defences (for review see [39]) and were found in the grapevine apoplast, as previously described for other species after stress perception [40-43]. However in this study, plants were not challenged with pathogens. In such context, the function of identified PR proteins could be attributed to a preformed defence, creating an environment in the apoplast that is harmful to pathogens.

Among all identified PR proteins, osmotin-like proteins (spots n° 33, 34, 36, 49, 160, 161, 163, 173, Figure 1B) and thaumatin-like proteins (spots n° 162, 177, Figure 1B), due to their redundancy and their relative spot intensities, were the major PR proteins found in the AF, representing 19.6% and 1.4%, respectively, of the overall AF proteins detected on gels. They both belong to the PR-5 family [44]. Osmotins have also been identified in the rice AF but not in the AF of alfalfa, poplar or maize [8,16,20,29]. Osmotin-like proteins, representing at least one fifth of the proteome in the AF, may play a crucial role in the grapevine apoplast without any pathogen attacks as it has been shown for drought stress [45,46].

Chitinases (spots n° 19, 31, 44, 55, 59, 65, 121, 122, 125, 127, 128, 134, 135, 140, 141, 142, 144, 147, 148, 149, 155, 175, 176, Figure 1B) and glucanases (spots n° 22, 24, 27, 39, 40, 41, 42, 45, 46, 47, 61, 63, 64, 136, 167, 168, 169, Figure 1B) are also highly present in the AF, respectively 10.3% and 6.7% of the overall AF proteins detected on gels. They are well characterized for their role in plant defences by degrading fungal cell walls [47-49]. Identification redundancies among all these spots suggest a high rate of PTM in chitinases and glucanases, as it has been shown in wine in which several chitinases were found to be glycosylated [50]. Presence of PTM on apoplastic chitinases and thaumatin-like proteins has also been suggested for tobacco and alfalfa [16,38] and phosphorylation sites have been described on poplar apolastic thaumatin-like proteins [20]. Moreover, Pechanova *et al.* have shown that 30% of poplar AF proteins are putatively glycosylated [20]. Glucanases, chitinases and thaumatin-like proteins are also the PR proteins most commonly found in AF of other plant species. Moreover, no changes in PR protein levels were reported in the AF proteomic analyses from stressed plants such as oligogalacturonide-treated Arabidopsis or pathogen-infected soybean [19,29,30]. These studies suggest that a large amount of PR proteins are already constitutively expressed in the AF.

Other less common PR proteins have been identified in the AF of other plant species but not systemically in all of them. For example the homologous NtPRp27 protein from tobacco found in grapevine AF with 82% homology (Table 1, spot 152) was also identified in poplar with 72% homology [20] and in alfalfa [16]. PR10 proteins were also found in alfalfa but not in poplar, rice or maize [8,16,20,29]. Interestingly, to our knowledge, the PR1 protein has been related to biotic or abiotic stress responses but never identified in AF of unstressed leaves from other plant species. All together these data suggest that the spectrum of PR proteins identified in the grapevine AF is larger that those previously observed in the AF of other various plant species.

#### Proteases

Various proteases were identified in the AF and they represented the second largest listed group with 16% of the overall AF proteins in grapevine leaf (Figure 5). Although this protein family is systematically found in other AF plant species, this large amount of proteases in AF grapevine could be related to the absence of protease inhibitors. Indeed no protease inhibitors have been found in grapevine AF while some protease inhibitors are found in maize, poplar or tobacco AF [8,20,38].

Proteases exhibit a broad spectrum of physiological roles, and increasing evidence indicates that they play a key role in response to stress, leading to a harmful environment for pathogens [12,51]. Among the proteases, 2 classes could be distinguished (i) subtilisin-like serine proteases (including subtilisin-type proteases, cucumisin proteases, xylem serine proteinases and serine carboxypeptidases) and (ii) aspartic proteases.

Subtilisin-like serine proteases (spots n° 3, 4, 5, 68, 69, 70, 71, 72, 80, 81, 82, 83, 89, 90, 94, 95, 110, 119, Figure 1B) are known to activate plant defence related genes and could participate in building physical barriers against pathogens [52]. Subtilisin-like proteases have been found in *V. vinifera* xylem sap [53] and *V*. *vinifera* cell culture secretome [54].

Aspartic proteases (spots n° 14, 15, 16, 17, 18, 48, 57, 99, 102, 103, 115, 117, 118, 119, 120, 123, 124, 126, 129, Figure 1B) have also been proposed to be involved in the direct defence against pathogens and in signalling processes by releasing systemic signal molecules after fungal protein degradation [51,55].

#### Cell wall metabolism and remodelling enzymes

Many glycoside hydrolases were found in the grapevine AF and classified as cell wall metabolism proteins in Table 1. The proteins involved in the cell wall metabolism are consistently found in the AF of other species studied so far. Quantitatively, proteins involved in the cell wall metabolism represent 12% of the total amount of grapevine AF proteins which is similar to the amount found in tobacco (15%) but lower than those found in rice (26%), poplar (32%) or maize (58%) [8,20,29,38]. However, these values have to be considered with caution because according to different studies, some protein families are sometimes also included in the cell wall metabolism, like oxidases in rice or peroxidases in poplar and maize [8,20,29]. Glycoside hydrolases are involved in metabolizing various carbohydrate compounds present in plant cell wall polysaccharides. They could also participate in glycan and glycolipid metabolism, energy mobilization, defence, symbiosis, signalling, as well as secondary plant metabolism and plant development [56]. Glycoside hydrolase genes have been reported to be induced during viral infection and development in grapevine [57,58]. They interact with hemicellulases and pectic enzymes to hydrolyze hemicelluloses and pectins and participate in cell wall structural modifications [59]. Moreover, it should be noted that in grapevine, glucosidases and galactosidases are the two most represented protein families in cell wall metabolism, as shown in almost all previous studied AF proteomes.

#### Other proteins with possible functions in plant defences

Further proteins related to plant defence reactions against biotic and abiotic stresses were identified in the grapevine AF. Peroxidases (spots n° 20, 21, 23, 25, 26, 29, 60, 62, 75, 76, 105, 106, 131, 158, Figure 1B) found in the AF are ubiquitous proteins, which could participate in broad functions in plants such as lignification, auxin catabolism, wound healing and defence against pathogen infection [60]. Peroxidases are commonly found in AF and are related to cell wall metabolism in poplar and maize, oxidoreduction in alfalfa [16] or stress in oilseed rape, poplar and maize [8,20,24]. Superoxide dismutase (spot n° 165, Figure 1B) shown to be secreted [37] is involved in the production of H_2_O_2_, one of the first lines of plant defence [61]. Serpin-like proteins (spot n° 127, Figure 1B) are able to inhibit serine protease targets and thus could interact with other proteins found in the apoplast [62]. Their function in plant defence processes could be linked to a protective effect against exogenous proteolytic attack (for review see [63]). Heparanase (spots n° 8, 10, 11, Figure 1B) already found in poplar and maize AF [8,20] is indirectly involved in H_2_O_2_ degradation. At the same time, it generates phenolic compounds that may be used for cell wall fortification [64,65]. To our knowledge it is the first time that a heparanase homolog and a serpin-like protein are reported in grapevine.

## Conclusions

The VIC method has been optimized to allow protein recovery from grapevine AF suitable for 2D-PAGE analyses. The large-scale proteomic analysis presented in this study established a well-defined proteomic map of whole leaf and leaf apoplastic soluble proteins. These two proteomic maps have been released in the public World-2DPAGE database to be used as interactive reference maps. To our knowledge, this is the first detailed proteome study of the grapevine apoplastic fluid providing a comprehensive overview of the most abundant proteins present in the grapevine apoplast. This proteome map of the leaf AF of *Vitis vinifera* should represent an essential complement to available genomic tools and should prove useful for systemic high-throughput studies on molecular processes in the apoplast such as molecular interaction between grapevine and its natural pathogens. Indeed, most of proteins found in the grapevine AF could have a clear role in stress responses, in cell wall metabolism and remodelling. Such data confirm that the apoplast has a major role in grapevine preformed defences.

## Methods

### Plant material

Vegetative cuttings of *V. vinifera* L. (cv. Chardonnay clone 7535) were obtained from healthy pruned canes of grapevine (Vranken Pommery, Reims vineyard, France) [66]. Cuttings were planted in 0.5 L pots containing loam and transferred to a control chamber at 20/26°C (night/day) with a 16 h light period (500 μmol. m^-2^.s^-1^) and relative humidity of 70%.

### Protein extractions

Apoplastic and whole leaf proteins were precipitated by trichloroacetic acid (TCA)/acetone and purified by phenol-based extraction [67,68].

Apoplastic fluids were collected by an adapted VIC method [11,12]. Proteins were extracted from 75 g of 10 week-old grapevine cuttings fully expanded leaves from the middle of the green shoots. Four biological repetitions of 120 cuttings each were performed and leaves were randomized. Since grapevine leaves are waxy and not very pulpy, increasing the difficulty of infiltration, leaves were cut into pieces of 1 cm^2^ to increase the accessibility for the infiltration buffer. Leaves were rinsed two times in ice-cold ddH_2_O in order to prevent contamination deriving from other cell compartments. Leaf pieces were dried by quickly blotting between two sheets of soft paper towel. Leaf pieces were then infiltrated with ice-cold infiltration buffer: 150 mM Tris–HCl, pH 8.5, containing 6 mM CHAPS in a vacuum chamber. The composition of the infiltration buffer was designed to facilitate the protein solubilization and to preserve as much as possible the plasmalemma integrity [7,8]. Infiltration was carried out 3–4 times within 10 min until leaves became glassy in appearance. Infiltrated leaf pieces were removed from the infiltration solution, washed twice with ddH_2_O to remove excess buffer and quickly dried again by blotting.

To collect AF, leaf pieces were arranged in bundles in a nylon mesh filter (diameter 6 μm). The bundles were placed in a 20 mL syringe in a 50 mL Falcon tube and centrifuged at 4°C at 7,500 g for 30 min.

Apoplastic soluble proteins were precipitated in 2 vol of 10% (w/v) TCA in acetone overnight at −20°C and pelleted by centrifugation at 10,000 g for 5 min.

For whole leaf soluble protein extractions, fully expanded leaves from the middle of vegetative grapevine green shoot cuttings were ground under liquid nitrogen. Two independent experiments of 40 cuttings each were performed and leaves were randomized. Extractions were carried out on 100 mg of ground plant tissue. After adding 1 mL of ice-cold acetone, samples were vigorously shaken and centrifuged at 10,000 g for 5 min. Supernatants were discarded and 1 mL of fresh ice-cold acetone was added. After shaking vigorously, the washing step was repeated, and 1 mL of ice-cold 10% (w/v) TCA/acetone was added to the pellet. A pipette tip was used to break up the pellet followed by a sonication step on ice (10 min) and subsequent centrifugation (10,000 g for 5 min) to pellet proteins.

Apoplastic and whole leaf soluble proteins pellets were washed once with ice-cold 10% (w/v) TCA/acetone and two times with ice-cold 80% (v/v) acetone. After the last centrifugation step, the residual acetone was carefully removed. Wet pellets were resuspended in 0.8 mL of dense SDS solution (100 mM Tris–HCl, pH 8, containing 30% (w/v) sucrose, 2% (w/v) SDS, 5% (v/v) 2-Mercaptoethanol). Then 0.8 mL phenol was added at room temperature (RT), samples were vortexed for 1 min and subsequently centrifuged at 10,000 g for 5 min (RT). The phenolic fraction was collected in fresh tubes. After addition of 5 volumes of ice-cold MeOH/0.1 M ammonium acetate, proteins were precipitated overnight at −20°C. Proteins were centrifuged (10,000 g, 10 min, 4°C) and the resulting pellet was washed 2 times with ice-cold MeOH/0.1 M ammonium acetate and 2 times with ice-cold 80% (v/v) acetone. The final pellet was air-dried and stored at −80°C. For 2D-PAGE analysis, pellets were dissolved in IEF rehydration buffer (2 M thiourea, 7 M urea, 2% (w/v) CHAPS, 0.5% (v/v) ampholyte (Invitrogen, Karlsruhe, Germany), 0.002% (w/v) Bromophenol Blue and 0.02 M DTT). An aliquot was used for protein quantification using the Bradford assay according to manufacturer’s instructions (Bio-Rad, Munich, Germany) with BSA as a standard.

### Protein profiling

For analytical 2D-PAGE separations, samples containing 250 μg proteins in rehydration buffer were applied on IPG strips (18 cm, 3–10 non-linear pH gradient, GE Healthcare Bio-Sciences AB, Uppsala, Sweden) by passive rehydration for 14 h. The first dimension was run on a Bio-Rad Protean IEF cell system (Bio-Rad, Munich, Germany) according to the manufacturer’s instructions for the recommended voltage ramp protocol (conditioning step: 250 V for 15 min, voltage ramping to 10,000 V during 3 h). After reduction and alkylation performed according to manufacturer’s instructions, the second dimension was run on an Ettan Dalt Six electrophoresis cell (GE Healthcare Bio-Sciences AB, Uppsala, Sweden) using 12% (v/v) polyacrylamide SDS-PAGE gels. Gels were run in a Tris-Glycine SDS buffer system at 40 mA per gel for approximatively 7 h and subsequently stained with Coomassie PAGE Blue (Fermentas, St. Leon-Rot, Germany) according to the manufacturer’s instructions. Spot detection and subsequent analysis was performed using the software Delta2D (Decodon, Greifswald, Germany) with default settings. Images were first aligned using the “group warping strategy”. Within both AF sample and total leaf sample, a master gel was selected to which all other gels in that sample were aligned. Following this, the master gels from the four replicate were aligned. All gel images were then fused to produce a single synthetic image containing all spots detected across the entire experiment. Protein spot volumes were normalized by dividing the individual spot volume by the sum of all spot volumes in that gel. After gel scanning, spots were visually selected and robotically punched from Coomassie stained gels for subsequent analysis using the Proteineer spII Spot picking robot (Bruker Daltonics, Bremen, Germany). Only the spots present in each replicate were further analysed. The reproducibility of the 2-D protein profiles was confirmed by carrying out 4 independent biological experiments.

### In-gel digestion of proteins and sample preparation for mass spectrometry analysis. MALDI data acquisition and database searching

Coomassie-stained protein spots were visually selected and then robotically excised and digested using the Proteineer spII and dp systems (Bruker Daltonics, Bremen). The resulting peptide mixtures were spotted as an HCCA suspension on AnchorChip TM targets by Proteineer dp robot for subsequent MALDI-TOF/TOF MS analysis. PMF data were collected on an UltraflexIII MALDI TOF/TOF mass spectrometer (Bruker Daltonics). Following a first round of database searching and on-target recrystallization of the sample spots, MS/MS spectra were collected on selected precursors (see Additional file 3 for detailed information). LIFT-MS/MS spectra were also collected on selected precursors in order to confirm PMF based identifications and to further elucidate any unexplained peak [69]. Both MS and MS/MS data were used to search on the release non-redundant NCBI database (http://www.ncbi.nlm.nih.gov) using MASCOT (http://www.matrixscience.com).

### Western-blot analysis

Twenty *μ*g of protein extracts were separated by SDS-PAGE on a 4-20% precast gel (Amersham Biosciences), transferred to polyvinylidene difluoride (PVDF) membranes for 7 min using IBlot gel transfer system (Invitrogen). PVDF membranes were incubated for 1 h with TBST (20 mM Tris–HCl, 500 mM NaCl at pH 7.5, 0.05% (v/v) Tween-20) containing 3% (w/v) of powdered milk. Membranes were incubated for 1 h with antibodies diluted in TBST with powdered milk (1:10000 for H^+^-ATPase and 1:5000 for Large subunit RuBisCo, RbcL). The polyclonal anti-H^+^-ATPase antibody was obtained from Dr. Marc Boutry (University of Louvain, Belgium)[26] and the anti-RbcL antibody was obtained from Agrisera Antibodies, Sweden. Goat anti-rabbit IgG horseradish peroxidase-conjugated was used as secondary antibody (1:10000) (Biorad) and the reaction was revealed after 5 min for RuBisCo and 30 min for H^+^ATPase by fluorography according to the manufacturer’s protocol (ECL; SuperSignal® west pico chemiluminescent substrate, Pierce).

### Bioinformatic analysis

Description of non-described amino acid sequences was taken up by Blast-p [22] (http://blast.ncbi.nlm.nih.gov/Blast.cgi).

Peptide p*I* and *M*_r_ values were theoretically determined using the Compute p*I*/*M*_r_ tool on the ExPASY Molecular Biology Server (http://expasy.org/tools/pi_tool.html) [70].

Gene ontology (GO) annotations (http://www.geneontology.org) were obtained by mapping GI numbers of the NCBI non-redundant protein database to the existing annotations of characterized proteins with GORetriever, provided by the AgBase web server (http://www.agbase.msstate.edu). Plant GO-slims identification for protein molecular functions were obtained from all GO terms associated with the protein annotation list by using the GOSlimViewer from the AgBase web server [27].

The secretion of apoplastic proteins was predicted using TargetP (http://www.cbs.dtu.dk/services/TargetP) [28], and SecretomeP (http://www.cbs.dtu.dk/services/SecretomeP) analysis to predict classically and non classically secreted proteins respectively [34,71]. A Transmembrane Hidden Markov Model (TMHMM) analysis was performed using the TMHMM server v.2.0. (http://www.cbs.dtu.dk/services/TMHMM) to predict transmembrane proteins [31,32].

## Abbreviations

AF: Apoplastic fluid; GO: Gene ontology; FW: Fresh weight; LSPs: Leaderless secreted proteins; PR: Pathogenesis-related; PTM: Post-translational modification; TCA: Trichloroacetic acid; TMHMM: Transmembrane Hidden Markov Model; VIC: Vacuum-infiltration-centrifugation.

## Competing interests

The authors have declared no conflict of interest.

## Authors’ contributions

BD conducted grapevine growth experiments, optimized the apoplastic fluid extractions, carried out the protein extraction, protein characterization by MALDI-TOF/TOF MS and analyzed the MS data, participated to the interpretation of the results and prepared the manuscript. TC analyzed the MALDI-TOF/TOF MS data and LIFT-MS/MS data. NB performed the bioinformatic analyses. AC helped with the apoplastic fluid extractions. AH conducted the protein samples preparation and 2D-PAGE. FB suggested the conception of the experimental design. CC took part in the critical revision of the manuscript. JS made the mass spectrometry facilities available and participated in the revision of the manuscript. PJ contributed to the interpretation of the results and participated to the writing of the manuscript. SC coordinated the experiments, participated to the interpretation of the results, wrote and edited the manuscript. All authors have read and approved the final manuscript.

## Supplementary Material

Additional file 1 **Data on protein identification from whole leaf sample.** Table shows the data related to gel image, MS and MS-MS analyses, prediction tools and blast-P search performed on all identified proteins from whole leaf sample. Click here for file

Additional file 2 **Data on protein identification from apoplastic fluid.** Table shows the data related to gel image, MS and MS-MS analyses, prediction tools and blast-P search performed on all identified proteins from apoplastic fluid sample. Click here for file

Additional file 3 Supplemental information on protein identification by MS and MS-MS analyses. Click here for file
