# Supplementary material for: Large-scale proteomic analysis of the grapevine leaf apoplastic fluid reveals mainly stress-related proteins and cell wall modifying enzymes
Source: BMC Plant Biol. 2013 Feb 8;13:24. doi: 10.1186/1471-2229-13-24 (PMC3640900; doi:10.1186/1471-2229-13-24)
Supplement: Additional file 3 — Supplemental information on protein identification by MS and MS-MS analyses. [file 1471-2229-13-24-S3.doc]

**Supplemental Information**

Data collection:

PMF –

Number of shots: 1200

Mass Range: 900 – 5000 m/z

MS/MS:

Parent Ion Isolation Assessment:

Automatic Precursor intensity optimization – 100 shots – Peak width < 0.8 m/z

Number of shots: 300 satisfactory spectra at final laser intensity

Fragmentation:

Relative Laser Intensity Boost: 45%

Precursor Ion Suppression

Number of shots: 800

Mass range: Precursor-dependent

Baseline substraction: Tophat

Smoothing:

PMF:Chemical Noise 0.15 m/z

MS/MS:Savistky/Golay 0.15m/z 4 cycles

Peak picking:

Algorithm: SNAP

PMF: 200 peaks, sig/noise>4

MS/MS: 200 peaks, sig/noise>3

Centroid width 0.2m/z, height 90%

Mascot searching:

Number of tryptic miscleavages allowed: 1

Peptide tolerance for PMF 50 ppm 150 ppm (without post calibration)

Peptide Mass Tolerace for MS/MS precursor 0.4 Da fragments 0.4 Da

Fixed mods – Carbamidomethyl Cystein

Variable modes – Methionine Oxidation

Mascot score thresholds reflecting the 95% certainty cutoff

NCBInr (no taxonomic restriction)

MS:82    MSMS:53

NCBInr (restricted to green plants)

MS:69    MSMS:41
